# Cross-Domain Transfer of EEG to EEG or ECG Learning for CNN Classification Models

**DOI:** 10.3390/s23052458

**Published:** 2023-02-23

**Authors:** Chia-Yen Yang, Pin-Chen Chen, Wen-Chen Huang

**Affiliations:** Department of Biomedical Engineering, Ming-Chuan University, Taoyuan 333321, Taiwan

**Keywords:** cross-domain transfer learning, electroencephalography (EEG), electrocardiography (ECG), convolutional neural network (CNN), seizure prediction, sleep staging

## Abstract

Electroencephalography (EEG) is often used to evaluate several types of neurological brain disorders because of its noninvasive and high temporal resolution. In contrast to electrocardiography (ECG), EEG can be uncomfortable and inconvenient for patients. Moreover, deep-learning techniques require a large dataset and a long time for training from scratch. Therefore, in this study, EEG–EEG or EEG–ECG transfer learning strategies were applied to explore their effectiveness for the training of simple cross-domain convolutional neural networks (CNNs) used in seizure prediction and sleep staging systems, respectively. The seizure model detected interictal and preictal periods, whereas the sleep staging model classified signals into five stages. The patient-specific seizure prediction model with six frozen layers achieved 100% accuracy for seven out of nine patients and required only 40 s of training time for personalization. Moreover, the cross-signal transfer learning EEG–ECG model for sleep staging achieved an accuracy approximately 2.5% higher than that of the ECG model; additionally, the training time was reduced by >50%. In summary, transfer learning from an EEG model to produce personalized models for a more convenient signal can both reduce the training time and increase the accuracy; moreover, challenges such as data insufficiency, variability, and inefficiency can be effectively overcome.

## 1. Introduction

Electroencephalography (EEG) is often used to evaluate several types of neurological brain disorders, such as epilepsy, dementia (e.g., Alzheimer’s disease), mental illness (e.g., depression), sleep disturbance, and unexplained headaches (e.g., intracranial hematoma) [1]. As artificial intelligence techniques have improved, many researchers have used machine-learning or deep-learning technology to identify or classify physiological signals [2,3,4] to reduce the burden on doctors and the time patients spend waiting for their diagnosis. Although machine learning is a mature field, with most algorithms, domain knowledge still needs to be applied for the feature selection [5]. By contrast, in deep-learning, useful features are automatically extracted, simplifying data preprocessing and improving recognition performance. For example, Shoeibi et al. [6] compared the performance of several conventional machine-learning methods—including support vector machine (SVM), k-nearest neighbors, decision tree, naïve Bayes, random forest, extremely randomized trees, and bagging—with that of three deep-learning architectures—the convolutional neural network (CNN), long short-term memory (LSTM), and one-dimensional (1D) CNN-LSTM—in schizophrenia (SZ) diagnosis based on z-score-normalized EEG signals from 14 subjects without and 14 patients with SZ. Bagging classification obtained the highest accuracy from the machine-leaning models (81% accuracy); the best deep-learning algorithm, the 1D-CNN-LSTM model, achieved a substantially superior accuracy of 99%.

However, the application of deep-learning requires the collection of a large dataset and substantial training time. In practice, the accuracy of models that have been well-trained often decreases substantially when the models are applied to new data. For example, Cimtay and Ekmekcioglu [7] selected a pretrained CNN model, InceptionResnetV2, for classifying emotions from EEG data. In their one-subject-out binary classification tests on the SJTU Emotion EEG Dataset (SEED), InceptionResnetV2 achieved a mean accuracy of 82.94%; however, the mean cross-dataset prediction accuracy of the model trained on SEED and tested on the Loughborough University Multimodal Emotion Dataset was only 57.89%. That means developing and training a bespoke model for each patient would require an excessive investment of time and resources.

Furthermore, many sleep monitoring studies have input multichannel EEG data to deep-learning models successfully. However, for clinical use, multichannel EEG must be performed by a professional. If such signals were to be collected using a wearable device at home, various factors would have to be considered, including long-term data storage, easy operation by a nonprofessional, and user comfort. Hence, many researchers have begun to investigate the potential of using other physiological signals—such as electrocardiogram (ECG), respiration, or blood oxygen—for sleep assessment. For example, Urtnasan et al. [8] used a deep convolutional recurrent model for the automatic scoring of sleep stages on the basis of raw single-lead ECG data from 112 subjects. They achieved an overall accuracy of 74.2% for five classes and 86.4% for three classes. Although they concluded that ECG can be used for at-home sleep monitoring, effectively improving the low accuracy of this method would be challenging.

In recent years, researchers have applied transfer learning in attempts to overcome these challenges (e.g., [9,10]). In transfer learning, the knowledge of a trained model, such as its features and weights, are input to a new model for further use. That means it reuses a pre-trained model for a new problem. Many implementations are to start from a pre-trained model, remove/freeze task-specific top layers, and fine-tune bottom layers of the new data. Here, the pre-trained models are partially transferred since only parameters in the bottom layers are transferred. Some examples of pre-training models in fine-tuning include AlexNet, ResNet, and VGG-16 [11]. This can greatly reduce not only the training data required but also the computing resources and time required for training a new model. For example, Zargar et al. [12] combined three ImageNet CNNs with three classifiers for predicting seizures. The Xception convolutional network with a fully connected (FC) classifier achieved a sensitivity of 98.47% for 10 patients from a European database, and the MobileNet-V2 model with an FC classifier trained on only one patient’s data but tested on six other patients achieved a sensitivity of 98.39%. Their study demonstrated the feasibility of the cross-patient application and performance improvements enabled by transfer learning. One interesting application of transfer learning is cross-signal transfer learning, in which a pretrained model with one type of signals is transferred to another, completely different type of signals. However, cross-domain transfer learning is rarely applied in the medical literature. Bird et al. [13] attempted to use unsupervised transfer learning to adapt a multilayer perceptron and CNN network for EEG classification to electromyographic (EMG) classification. Their results revealed that if only EEG or EMG was used to train the model, the accuracy was 62% or 84%, respectively. However, EEG to EMG transfer learning (i.e., EEG pretrained weights were used as the initial weight distribution for the EMG classification models) and EMG to EEG transfer learning achieved accuracies of 85% and 93%, respectively. Hence, EEG to EMG transfer learning did result in a higher initial classification accuracy than using EMG alone; however, the improvement was lower than that of EMG to EEG transfer learning. This result demonstrated the possibility of using cross-domain transfer learning for different biosignals to reduce both the complexity of the models and the difficulty and tediousness of signal collection.

EEG can be used to detect brain abnormalities and provides an effective basis for patient evaluation. However, the method has many practical challenges. By using transfer learning, the aforementioned problems—time-consuming model training, low accuracy on novel data, and insufficient training data—might be effectively solved. Therefore, this study attempted to apply transfer learning to EEG-based classification to explore the effectiveness of various cross-domain training methods for improving recognition performance. Two simple experiments were performed for the verification of the proposed methods: (1) in Experiment 1, a seizure prediction system for detecting interictal and preictal periods was developed by using a patient-specific/cross-dataset transfer learning strategy. The preictal period was defined as 20, 30, or 40 min before a seizure. Epilepsy is a chronic neurological disease caused by abnormal brain electrical activity; it influences the behavior, movement, sensory perceptions, or cognition to negatively affect work, daily life, and social relationships [14]. An early seizure warning could greatly reduce the danger to and harm experienced by patients with epilepsy. In the experiment, a general epilepsy prediction model based on a CNN was first developed and then adapted for particular patients by using transfer learning to fine-tune parameters with the goal of reducing the model development time and improving the results for each patient. (2) In Experiment 2, a sleep staging system for detecting the five sleep stages was developed by using a cross-signal transfer learning strategy. Collecting ECG signals during sleep is easier and more convenient than collecting EEG signals; however, ECG models typically have lower accuracy. Hence, in the experiment, a CNN-based sleep staging model for EEG was first developed and validated; the EEG model was then converted into an ECG model and fine-tuned in an attempt to reduce the required number of training samples for the ECG model and achieve higher accuracy. CNN is a common type of neural network model used in deep-learning. Because of its automatic detection of visual features, CNN is widely used in image segmentation and classification. This main advantage is also suitable when applied to EEG raw data for a variety of recognition purposes [15].

## 2. Materials and Methods

### 2.1. Experiment 1

#### 2.1.1. Datasets

EEG data were downloaded from two datasets: the Siena Scalp EEG database and Zenodo database. From the Siena Scalp EEG database, EEG signals for 13 patients with epilepsy (mean ± standard deviation age 42.6 ± 13.8 years) were obtained; one patient included in the database had data of insufficient length, so these data were excluded. The record duration was 9 h 17 min ± 5 h 39 min [16,17]. From the Zenodo dataset, EEG signals were obtained for 14 patients with epilepsy (age 17.4 ± 9.6 years), excluding one as well, with a record duration of 7 h 55 min ± 4 h 15 min [18]. For each patient, the diagnosis of epilepsy and classification were made by a doctor. All patients provided written informed consent approved by the Ethics Committee of the University of Siena.

#### 2.1.2. Data Acquisition

The EEG signals from both datasets were recorded using a Video-EEG with 29 channels in accordance with the International 10-20 system (i.e., FP1, F3, C3, P3, O1, F7, T3, T5, Fc1, Fc5, Cp1, Cp5, F9, Fz, Cz, Pz, FP2, F4, C4, P4, O2, F8, T4, T6, Fc2, Fc6, Cp2, Cp6, and F10) at a sampling rate of 512 Hz.

#### 2.1.3. Data Analysis

**Figure 1:**
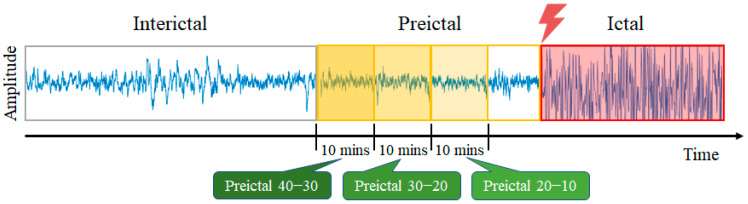
Illustration of four epileptic states in EEG signals.

EEG signals were preprocessed using MATLAB R2019a v9.6.0 in three steps: (1) all signals were detrended to remove means, offsets, and slow linear drifts over the time course; (2) the detrended signals were filtered using a 0.5–50 Hz bandpass filter; and (3) the global field power was computed over time for the filtered 29-channel signals using the formula [19]:

(1) $$GFP\left(t\right)=\sqrt{\overset{N}{\underset{i=1}{\sum }}{\left(x_{i}\left(t\right)-\bar{x_{t}}\right)}^{2}∕N}$$

where *t* is the time in milliseconds, *N* is the number of channels, $x_{i}$ is the value at time point *t*, and $\bar{x}$ is the mean value across channels at time point *t*. After preprocessing, the signals were truncated by using 10-s overlapping windows with 8 s of overlap and divided into four epileptic states: (1) seizure: the period after the previous seizure and before the current seizure with an interval of at least 50 min [13]. (2) Preictal 20–10: 20 min to 10 min before the seizure. (3) Preictal 30–20: 30 min to 20 min before the seizure. (4) Preictal 40–30: 40 min to 30 min before the seizure. A total of 12,222 samples were obtained for each state (Figure 1).

#### 2.1.4. Classification and Performance Evaluation

The CNN model was implemented using Python v3.8.8 on a personal computer with an Intel Core i7-10700K CPU, NVIDIA Quadro RTX 4000, and 64.0 GB of RAM running Windows 10 with CUDA 10.1. We modified the model of Wang et al. [20]; the model comprised four convolutional layers, five pooling layers, and three FC layers (Table 1).

**Figure 2:**
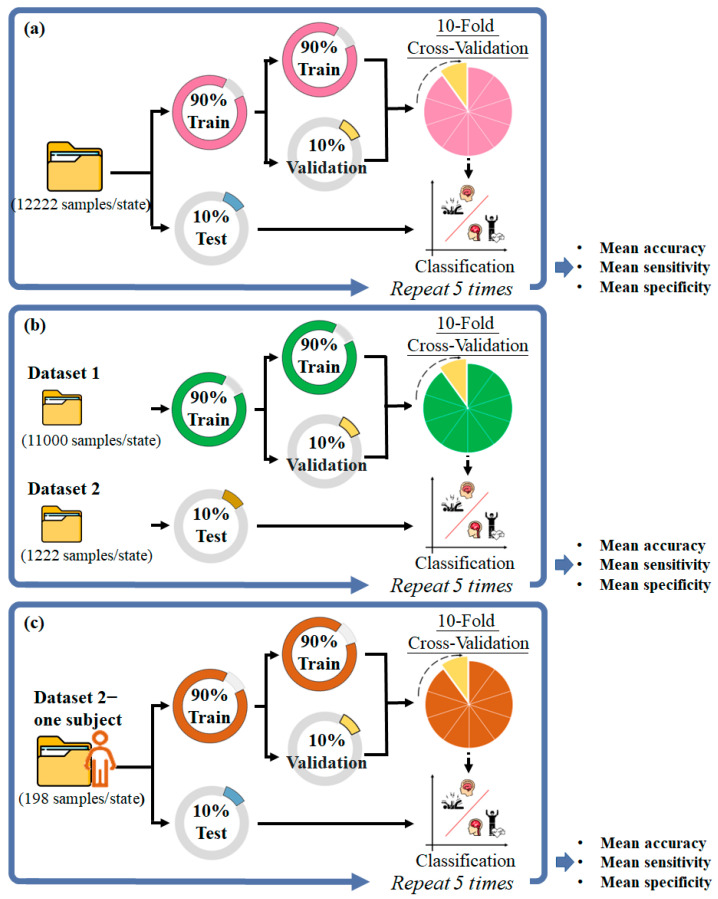
Scheme of the training process for a 10-fold cross-validation by using (**a**) recordwise, (**b**) subjectwise, and (**c**) patient-specific approaches.

Three approaches were used for training: recordwise, subjectwise, and patient-specific. For all approaches, 10-fold cross-validation was used to evaluate the trained models. The optimized model was then validated on the testing dataset by calculating its accuracy, specificity, and sensitivity. These processes were performed five times (Figure 2).

**Figure 3:**
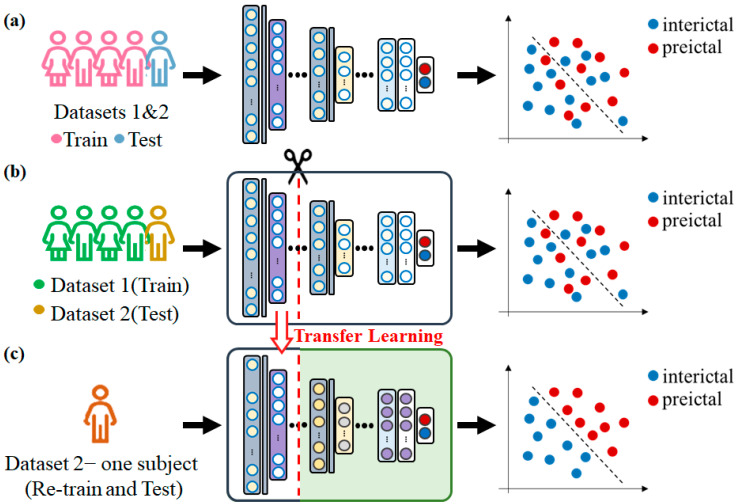
Basic procedure for the classification of preictal and interictal periods by using (**a**) recordwise, (**b**) subjectwise, and (**c**) patient-specific approaches.

In the recordwise approach, data from two datasets were randomly divided into two sets: 90% for training (approximately 11,000 samples per state) and 10% for testing (approximately 1222 samples per state). In the subjectwise approach, the Siena Scalp EEG data were used for training (11,000 samples per state), and the Zenodo data were used for testing (1222 trials per state). In the patient-specific transfer learning, the subjectwise-trained model was transferred to a model for the data of an individual subject in the Zenodo dataset. Subject data were randomly divided into training and testing datasets in a 90:10 ratio (178 and 20 samples per state, respectively). The first 12, 9, 6, or 3 layers were frozen (i.e., their weights were fixed) and the unfrozen layers were retrained for the individual. The performance of the models with various numbers of frozen layers was compared (Figure 3).

### 2.2. Experiment 2

#### 2.2.1. Datasets

We used EEG data downloaded from the Sleep Cassette subset of the Sleep-EDFX database [16,21], which consists of 153 polysomnographic (PSG) recordings. Seventy-eight healthy subjects (age = 58.8 ± 22.4 years) were included and the record duration was approximately 20 h, including the whole sleep period. The ECG data were downloaded from the Haaglanden Medisch Centrum (HMC) sleep staging database [16,22], which consists of 154 PSG files. A total of 154 patients with different sleep disorders (age = 53.8 ± 15.4 years) were included, and the record duration was 7–13 h.

#### 2.2.2. Data Acquisition

**Figure 4:**
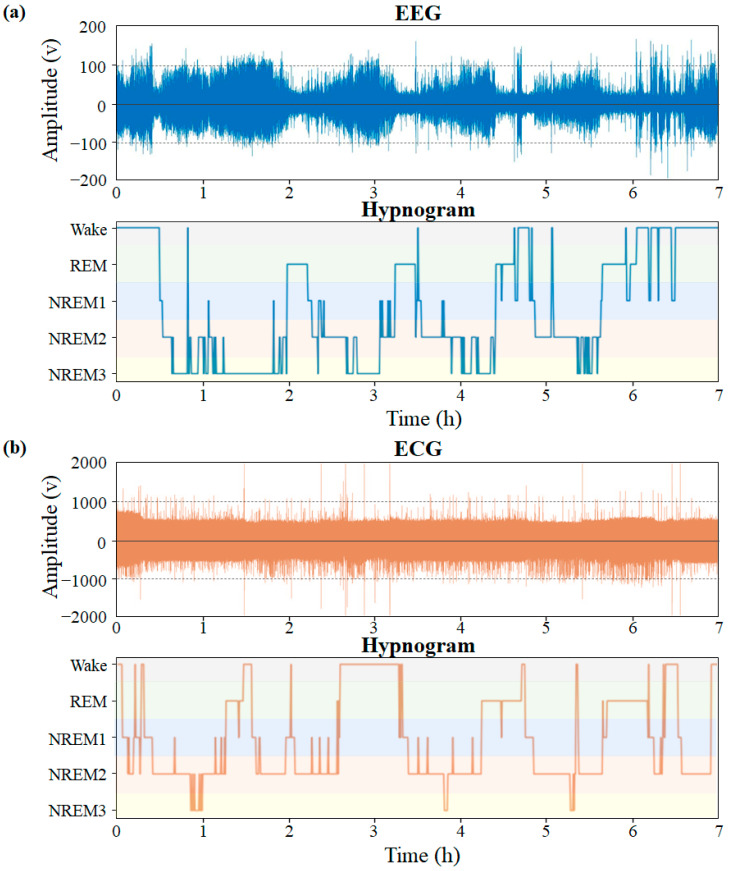
Examples of sleep recordings and hypnograms from the (**a**) EEG, and (**b**) ECG datasets.

EEG signals were recorded using a dual-channel (Fpz-Cz) cassette recorder at a sampling rate of 100 Hz. Each 30-s epoch was manually labeled by experts in accordance with the R&K standard [23] as belonging to one of six sleep stages: wake, S1, S2, S3, S4, or REM. We coded S1 as NREM1, S2 as NREM2, and combined S3 and S4 as NREM3 in accordance with the American Academy of Sleep Medicine (AASM) standard. ECG signals were recorded using a SOMNOscreen PSG recorder at a sampling frequency of 256 Hz. Each 30-s epoch was manually labeled by sleep technicians at HMC in accordance with the AASM standard (Figure 4).

#### 2.2.3. Data Analysis

EEG signals were preprocessed in two steps using MATLAB R2019a v9.6.0. First, all signals were detrended to remove means, offsets, and slow linear drifts over the time course. These detrended signals were then filtered using a 30-Hz lowpass filter. After preprocessing, the signals were truncated using 30-s windows with 22.5-s overlaps and categorized by sleep state. A total of 16,000 samples were obtained for each state. ECG signals were similarly preprocessed in two steps using MATLAB R2019a v9.6.0. All signals were first detrended to remove means, offsets, and slow linear drifts over the time course. These detrended signals were then filtered using a 0.5–40-Hz bandpass filter. After preprocessing, the truncation process was performed for the EEG signals. A total of 16,000 samples were obtained for each state.

#### 2.2.4. Classification and Performance Evaluation

**Figure 5:**
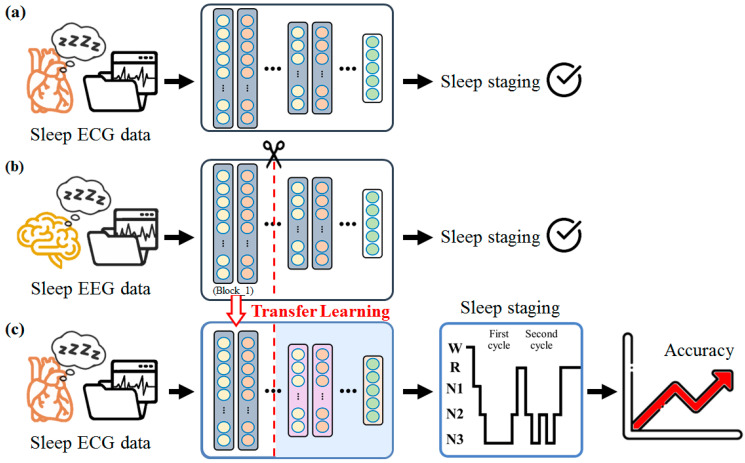
Basic procedure for the sleep staging classification in the (**a**) ECG model, (**b**) EEG model, and (**c**) EEG–ECG transfer learning model.

The CNN model was implemented using Python v3.5.4 running on a personal computer with an Intel Core i7-9700K CPU, NVIDIA Geforce RTX 2060, and 64.0 GB of RAM and running Windows 10 with CUDA 10.1. We modified the model of Jadhav and Mukhopadhyay [24]; the model comprised three blocks and two FC layers. Block_1 and block_2 each comprised two convolutional layers, two batch normalipyzation (BN) layers, and one pooling layer; block_3 comprised one convolutional layer, one BN layer, and one global pooling layer (Table 2). The EEG to ECG transfer learning was performed in three steps: (1) the construction of an EEG-based sleep stage model; (2) transfer of the trained EEG model to an ECG model; and (3) freezing of block_1–3, block_1–2, or block_1 (i.e., fixing the pretrained weights) and retraining of the unfrozen layers (Figure 5).

**Figure 6:**
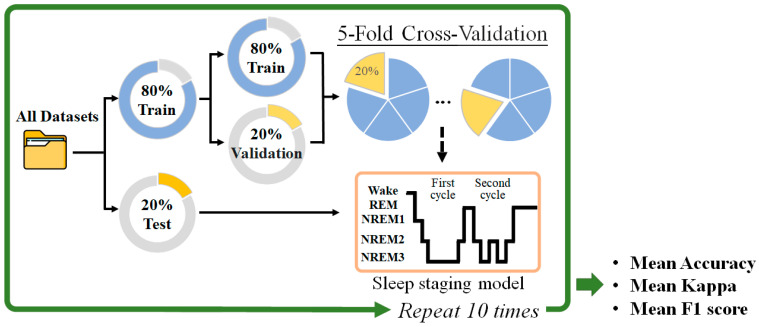
Scheme of the training process for a 5-fold cross-validation.

Data were randomly divided into two sets: 80% for training and 20% for testing. A five-fold cross validation was used to evaluate the trained models. The optimal model was then tested using the testing dataset and evaluated its accuracy, Cohen’s kappa, and the F1-score. These processes were performed 10 times (Figure 6).

## 3. Results

### 3.1. Experiment 1

The effectiveness of the three training approaches for establishing a CNN-based epilepsy prediction model was investigated. The results for the recordwise training (Table 1) revealed that the accuracy, sensitivity, and specificity for classifying interictal and preictal 20–10-, 30–20-, and 40–30-min states were all greater than 99%, 98%, and 99%, respectively; the training time for all three models was approximately 2 h. Hence, this training approach had an excellent performance, but the training was somewhat time-consuming.

The results for subjectwise training (Table 3) revealed that the accuracy, sensitivity, and specificity for the classifying of interictal and preictal 20–10-, 30–20-, and 40–30-min states were all greater than 82%, 84%, and 83%, respectively; however, the training time for all three models was still approximately 2 h. Hence, a comparison of the results for recordwise and subjectwise training revealed that if the novel subject data were not used for the model training, the test accuracy, sensitivity, and specificity decreased but the training time remained constant.

The results for patient-specific transfer learning (Table 4) differed from those for recordwise and subjectwise training. The models with 12 frozen layers that were used to classify interictal and preictal 20–10-, 30–20-, and 40–30-min states achieved metrics greater than 90%, 88%, and 95%, respectively, with training times of approximately 2 min. Models with nine frozen layers classifying interictal and preictal 20–10-, 30–20-, and 40–30-min states achieved metrics of 100%, >98%, and >96%, respectively, with training times of approximately 45 s. Those with six frozen layers achieved metrics of 100%, >97%, and >99% with training times of approximately 40 s, and those with three frozen layers achieved metrics of >96%, >94%, and >98% with training times of approximately 50 s, respectively. In summary, transfer learning training could be completed in approximately 1 min, and the accuracy, sensitivity, and specificity for most patients was high.

Specifically, the training time was shortest (~30 s) when freezing nine layers for the classification of interictal and preictal 40–30-min states for nine patients; freezing six layers resulted in the highest accuracy (except for patient 11 with 99.5%). Freezing 12 layers led to the longest training time (~2 min) and the lowest accuracy—which was even lower than when using the recordwise approach. The results thus indicated that transfer learning was superior to recordwise or subjectwise learning.

### 3.2. Experiment 2

Three CNN-based sleep staging models were established: the EEG model, the ECG model, and the EEG–ECG transfer learning model (Table 5). The EEG model for sleep stage classification achieved accuracy, Cohen’s kappa, and F1 scores of 92.67%, 0.908, and 92.69%, respectively; the training time (including five-fold cross validation) was approximately 1.5 h; the favorable Cohen’s kappa and F1 scores indicated that the model had favorable validity and reliability. The ECG model achieved accuracy, Cohen’s kappa, and F1 scores of 86.13%, 0.827, and 86.07%, respectively; the training time was still approximately 1.5 h. Finally, the EEG–ECG transfer learning model with block_1 frozen achieved metrics superior to the ECG-only model: 88.64%, 0.858, and 88.59%, with a lower training time of approximately 47 min. Freezing block_1 and block_2 or all three blocks resulted in lower scores than the ECG model; however, the training time was far shorter than that for the ECG model at approximately 17 min. Hence, the model with block_1 frozen (two convolutional layers, two BN layers, and one pooling layer) achieved both a higher performance and a lower training time than the ECG-only model.

**Figure 7:**
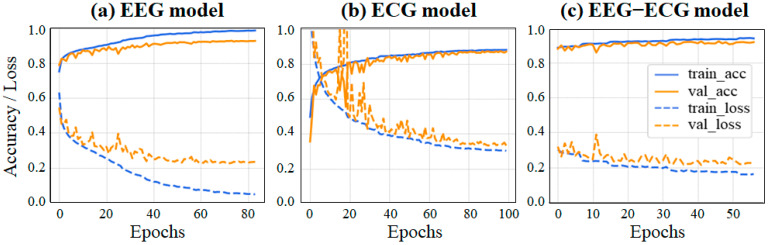
Accuracy (upper panel) and loss (lower panel) functions of the (**a**) EEG model, (**b**) ECG model, and (**c**) EEG–ECG model (frozen block_1).

Figure 7 illustrates the accuracy and loss functions of the EEG, ECG, and EEG–ECG models. An early stop strategy with a patience of 10 was implemented to terminate the training process. The validation accuracy and loss curve of the EEG model increased and decreased quickly, respectively. The validation accuracy and loss curves of the ECG model both fluctuated initially and then stabilized. For the EEG–ECG transfer learning model, the validation accuracy and loss curve were initially high and low, respectively, but slowly stabilized after fluctuating slightly. Overall, overfitting was not evident for any of the three models; hence, the training was judged to be effective.

**Figure 8:**
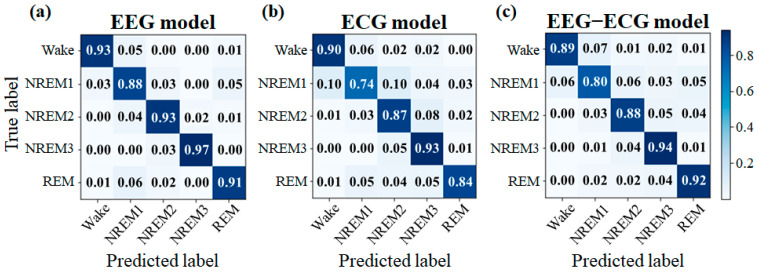
Confusion matrix of the (**a**) EEG, (**b**) ECG, and (**c**) EEG–ECG model (frozen block_1).

Figure 8 presents the confusion matrixes for the three sleep staging models. Except for NREM1, which was frequently misclassified as waking or NREM2, high classification accuracies were achieved. Hence, the three models achieved favorable classification results and no substantial imbalance was identified.

## 4. Discussion

### 4.1. Experiment 1

Recordwise training is a commonly used approach in initial deep-learning research. In training, data from all subjects in a database are randomly divided into a training set and testing set; each sample (from the same subject) is considered independent. For example, Acharya et al. [25] developed a computer-aided seizure diagnosis system to automatically distinguish the class of EEG signals (i.e., normal, preictal, or seizure) by using a 13-layer CNN model. They employed a dataset of 100 epochs for each of five healthy subjects and five patients with epilepsy, while 90% of the total data was set for training. Their model achieved an accuracy, specificity, and sensitivity of 88.67%, 90.00%, and 95.00%, respectively. Moreover, Wei et al. [26] proposed a long-term recurrent CNN for discriminating preictal from interictal states for seizure prediction. They similarly used a 9:1 ratio to divide the EEG data of each subject into training and test sets. Their seizure prediction model achieved an accuracy of 93.40%, prediction sensitivity of 91.88%, and specificity of 86.13%. In our experiment, all EEG samples from 27 patients with epilepsy in two datasets were mixed and were randomly divided into training and test sets (1222 samples per state). The classification accuracy, sensitivity, and specificity for interictal and preictal (regardless of period) states were all greater than 98%. These results indicate that the recordwise trained model is often only effective for classifying the used dataset, and its performance on novel data is poor [7].

Many studies have adopted subjectwise training for deep-learning models in which data from an individual are included in either the training or the testing set. This method better matches practical applications of the trained model to novel patients. However, the accuracy is an inevitable issue. In our experiment, we used EEG data from one dataset (Siena Scalp EEG database) for training, and EEG data from another dataset (Zenodo database) for testing. The accuracy decreased from 98% for the recordwise approach to only 84%; this may be attributable to the inter-person differences and the diversity of the data. This problem of a cross-subject domain shift has partly been addressed by some scholars. For example, Wang et al. [27] proposed a multiscale CNN, known as SEEG-Net, for evaluating drug-resistant epilepsy. They conducted cross-validation on a multicenter stereoelectroencephalography dataset by using the leave-one-group-out method and achieved an accuracy of 94.12% and 87.02% for the MAYO and FNUSA datasets, respectively; leave-one-subject-out cross validation on a private clinical dataset led to an accuracy of 93.85%. Although their proposed model performed highly in detecting pathological activity, it still has insufficient generalizability for practical applications.

The quality of EEG signals is affected by breathing, blinking, and swallowing during the measurement. In addition, individual differences may also affect evaluations based on these signals [28]. To avoid overfitting, deep-learning requires an enormous volume of training data and hence a long training time, delaying system development. Hence, we selected patient-specific transfer learning for retraining our model; specifically, data for a specific subject in the Zenodo dataset were used to fine-tune a model pretrained on the Siena Scalp EEG database. This method required a smaller amount of data, achieved high accuracy, and required little additional training time to produce the customized model. Layer-wise transfer learning is a commonly used approach in which some layers are frozen to decrease the training time. If a few layers are frozen, the model has high elasticity but requires a longer training time; by contrast, freezing many layers reduces the training time but often reduces the accuracy. Our experimental results indicated that a model with six frozen layers had a short training time (~40 s) and achieved the highest accuracy of nearly 100%. Freezing nine layers achieved a similar performance to freezing six layers; however, the imperfect results for patient 11 revealed that such a model may have insufficient elasticity to be applicable to all individuals. The optimal number of frozen layers may depend on the size of the training data [29]. Hence, for smaller datasets, training the FC layers alone is insufficient; some convolutional layers must also be trained to obtain a stable, accurate model.

Finally, we compared the accuracy rates of our model with those of models reported by other recent studies on epileptic seizure prediction using EEG data (Table 6). Dissanayake et al. [30] extracted Mel-frequency cepstrum coefficients (MFCCs) features from EEG signals and used them in a graph neural network (C-GNN) based on geometric deep-learning to predict epileptic seizures. Their subject-independent models were trained through a 10-fold cross-validation with over a 95% accuracy in both CHB-MIT and Siena databases. Zhao et al. [31] proposed a novel end-to-end model AddNet-SCL for seizure prediction based on EEG signals. They used a quasi-patient-specific method (i.e., 0.75 × (1−1/N), 0.25 × (1−1/N), and 1/N of a patient’s EEG data were used for the training, validation, and testing, respectively; where N was the number of seizure events) to conduct separate model training for each subject from CHB-MIT and Kaggle databases, and achieved 0.94 AUC and 0.831 AUC, respectively. Considering the robustness and generalization of the learning models, either training manner, i.e., subject independent or patient-specific, could achieve a high performance, while ours had the highest accuracy, specificity, and sensitivity. Furthermore, the use of raw EEG data in our experiment can facilitate the processes of data collection and processing and benefit future applications, bypassing the need for feature extraction or selection.

### 4.2. Experiment 2

Silveira et al. [32] used random forest to classify 106,376 single-channel EEG epochs from the Physionet public database into two- to six-state sleep stages. They computed the kurtosis, skewness, and variance of the coefficients decomposed through the discrete wavelet transform as classification features. The accuracy and Cohen’s kappa were >90% and >0.8, respectively, demonstrating that single-channel EEG is a feasible method of sleep staging. More recently, many studies have applied various deep-learning models for sleep staging with the goal of achieving automatic and accurate classification by avoiding manual feature extraction. For example, Yildirim et al. [33] developed a 1D-CNN model by using EEG signals from two public databases (Sleep-EDF and Sleep-EDFX) for the sleep stage classification. The accuracy of the model for five sleep classes on single-channel EEGs from the Sleep-EDF and the Sleep-EDFX databases was 90.83% and 90.48%, respectively. In our experiment, we also used the Fpz-Cz single-channel EEG signals from the Sleep-EDFX database for five-class sleep staging to train a modified 1D-CNN (10 layers in total; 9 layers fewer than in the model of [33]). The accuracy reached 92.67%, indicating that using fewer convolutional layers and max pooling instead of average pooling can slightly improve both the accuracy (~2%) and training efficiency. Although max pooling retains key sleep features in EEG, it ignores secondary features that may be effective for classification. By contrast, average pooling retains these features.

Due to the increasing prevalence of wearable biosignal sensors, many researchers have begun to study ECG sleep staging as an alternative to EEG staging. For example, Ebrahimi et al. [34] extracted features from ECG-derived respiration signals based on the R and S waves of the QRS complex, raw thoracic respiratory rate (R), and heart rate variability (HRV) and evaluated the performance of various signal combinations in an SVM automatic sleep staging model. Their best accuracy (89.32%) for classifying four stages—wake, Stage 2, slow wave sleep, and REM—was obtained when using HRV and R signals. Furthermore, Wei et al. [35] extracted 25 features from HRV and R signals and used LSTM for the two- to five-class sleep staging of patients with mental disorders, achieving accuracies of 89.84%, 84.07%, 77.76%, and 71.16% and Cohen’s kappa of 0.52, 0.58, 0.55, and 0.52, respectively, for the four classification tasks. These results indicate that increasing the number of classes decreases the performance; improving the accuracy requires the combination of various signals with different features. However, manual feature selection is time-consuming. Hence, some researchers have used deep-learning models for ECG sleep staging. For example, Tang et al. [36] used a CNN with gated recurrent units to classify sleep stages into four classes on the basis of single-lead ECG signals from the three public datasets SHHS2, SHHS1, and MESA. Their best accuracy and Cohen’s kappa were 80.6% and 0.70, respectively—a substantial improvement over previous attempts at cross-dataset classification. In our experiment, we used a CNN model for five-class sleep staging and an achieved average accuracy was 86.13%, which demonstrates that the model structure is effective for both EEG and ECG signals; the model achieved favorable performance for both signals, but the ECG model required more computational resources and training time.

Therefore, we applied transfer learning to improve the performance of the ECG model by basing it on the highly accurate EEG model. Freezing block_1 produced an EEG–ECG transfer learning model with an accuracy of 88.64%, a small improvement (~2.5%) compared with the ECG-only model. Radha et al. [37] trained an LSTM model to classify four-class sleep stages by using ECG data (292 participants, 584 recordings) and then transferred some of its weights to photoplethysmography (PPG) data (60 participants, 101 recordings) by using three transfer-learning strategies. The accuracy and Cohen’s kappa of the ECG–PPG model were 76.36% and 0.65, respectively—a substantial improvement over those of the PPG model (69.82% and 0.55). This result demonstrates the merit of transfer learning if similar data are reused. However, few studies have attempted cross-signal transfer learning. Phan et al. [10] trained two recurrent neural networks in the source domain (a large database; the Montreal Archive of Sleep Studies database in this case) and then fine-tuned them in the target domain (two small databases: the SurreycEEGrid database and the Sleep Cassette and the Sleep Telemetry subsets of the Sleep-EDFX database). The transfer learning achieved an improvement in accuracy of 1.5% for their SeqSleepNet+ network (78.5% for EEG-only to 80.0% for EEG-EOG) and 3.5% for their DeepSleepNet+ network (75.9% to 79.4%). These transfer-learning studies reveal that the knowledge transfer from the same or similar signals can considerably increase model performance; for different signals, however, it does not greatly increase the accuracy but substantially reduces the training time, in our case. Moreover, if too many layers are frozen (too much knowledge is shared), training the new model has a limited effect and the model may fit the data poorly, resulting in high-speed training but low performance.

Finally, we compared the accuracy rates of our model with those of models reported by other recent studies on sleep staging using EEG data (Table 7). Li et al. [38] proposed an EEGSNet model based on CNN and bi-directional LSTM (Bi-LSTM) to extract features from the EEG spectrogram and classify them into five sleep stages. They trained their model using a 20-fold or leave-one-out cross-validation according to the size of the datasets. The accuracies were 94.17%, 86.82%, 83.02%, and 85.12%, respectively, for the sleep-edfx-8, sleep-edfx-20, sleep-edfx-78, and SHHS datasets. Jadhav et al. [24] evaluated the raw EEG epochs, short-time Fourier transform (STFT), and stationary wavelet transform (SWT) in the same dataset (i.e., sleep-edfx-78) by using CNN models. Their subject-wise models were trained through a 20-fold cross-validation with over 83% accuracy. For the classification of five sleep stages, our model with fewer layers achieved a better performance and the direct use of raw EEG data in our experiment can be of benefit for fast diagnosis. Table 8 shows the comparison of our model with other recent closely related studies using ECG data. Urtnasan et al. [8] used a deep convolutional recurrent (DCR) model based on the CNN and a gated recurrent unit (GRU) for the automatic scoring of sleep stages. They trained and tested the model using the ECG signals of 89 subjects and 23 subjects, respectively, randomly selected from the dataset and achieved an overall accuracy of 74.2% for five classes and 86.4% for three classes. Tang et al. [36] pre-trained a model built on five CNN blocks, bi-directional GRU layers, and a fully connected layer with a dataset and then re-trained it with another dataset with an improvement of 20%. Considering the resources and time, they randomly sampled 100 subjects (70% for training and 30% for testing) from each dataset. There is still room for improvement in the effect of using ECG signals alone for classification. By using transfer learning from EEG to ECG, our model could classify more classes with a better performance, which demonstrates the feasibility of automatic sleep staging using ECG signals.

Our experiments have some limitations. First, the sample size was insufficient. Including more databases in the training and test sets would improve the reliability of the model. Second, temporal information was not considered. Automatic feature extraction coupled with time series training, such as CNN-LSTM, may be more effective.

## 5. Conclusions

This study attempted to apply cross-domain transfer learning for two EEG-based classification tasks—seizure prediction and sleep staging—to explore its effects on recognition performance.

In Experiment 1, binary classification models were trained using a recordwise approach to test the architecture of our model; this model achieved an accuracy, specificity, and sensitivity of >98%. Subsequent subjectwise training simulated practical applications in which the test and training data were independent; this model achieved an accuracy, specificity, and sensitivity of >82%. Due to this dramatic decrease in the model performance, cross-dataset transfer learning was used to train patient-specific models; the model with six frozen layers achieved an accuracy, specificity, and sensitivity of 100% for seven out of nine subjects and >97% for the remaining two; moreover, only 40 s of additional training time was required. By applying transfer learning, the model could learn the EEG characteristics of an individual to achieve personalized and accurate detection that could increase the practicality of seizure prediction.

In Experiment 2, transfer learning on different signal sources for five-class sleep staging prediction was attempted. The same modified model architecture was used to build EEG and ECG models. As expected, the accuracy, Cohen’s kappa, and F1-score (92.67%, 0.908, and 2.695%) of the EEG model were superior to those of the ECG model (86.13%, 0.827, and 86.07%). However, transfer learning produced an EEG–ECG model with an accuracy approximately 2.5% greater than that of the ECG model. Although this cross-signal transfer-learning method achieved little performance improvement, the training time was reduced by >50% compared with that for the ECG-only model, effectively reducing the computing resource consumption. Additional studies should be conducted regarding the challenges of knowledge transfer between different signals. To the best of our knowledge, this experiment is the first to demonstrate the feasibility of cross-signal transfer learning from EEG to ECG for sleep staging. EEG measurement is inconvenient and uncomfortable; hence, using ECG for sleep staging could enable practical applications, such as wearable devices employed for sleep analysis and recording sleep quality.

In summary, EEG can be used to detect brain abnormalities and provides an effective basis for patient evaluation. However, its limitations restrict its use in practice. Cross-domain transfer learning strategies may be able to overcome these problems for further specific uses, such as precision medicine, portable devices, or rare disease detection, in simple or original model structures.

## Figures and Tables

**Table 1:** Parameters of the CNN model for seizure prediction.

Layer	Type	Filter Size	# Filter	Stride	Output
conv1d_1	Conv1D	10	32	2	2556 × 32
batch normalization_1	Batch Normalization	-	-	-	2556 × 32
max_pooling1d_1	MaxPooling1D	3	1	1	2554 × 32
conv1d_2	Conv1D	10	64	2	1273 × 32
batch normalization_2	Batch Normalization	-	-	-	1273 × 32
max_pooling1d_2	MaxPooling1D	3	1	1	1271 × 32
conv1d_3	Conv1D	10	64	2	631 × 64
batch normalization_3	Batch Normalization	-	-	-	631 × 64
max_pooling1d_3	MaxPooling1D	3	1	1	629 × 64
conv1d_4	Conv1D	10	128	1	620 × 128
batch normalization_4	Batch Normalization	-	-	-	620 × 128
max_pooling1d_4	MaxPooling1D	3	1	1	618 × 128
global_average_pooling1d	GlobalAveragepooling	-	-	-	128
dense_1	Dense	-	-	-	256
dense_2	Dense	-	-	-	128
dense_3	Dense	-	-	-	2

Hyperparameters: optimizer = Adam, batch size = 128, learning rate = 0.0002 (reduce_lr: min_lr = 0.00001).

**Table 2:** Parameters of the CNN model for sleep staging.

Block	Layer	Type	Filter Size	# Filter	Stride	Output
Block_1	conv1d_1	Conv1D	5	16	1	2996 × 16
	batch normalization_1	Batch Normalization	-	-	-	2996 × 16
	conv1d_2	Conv1D	5	16	1	2994 × 16
	batch normalization_2	Batch Normalization	-	-	-	2994 × 16
	average_pooling1d_1	AveragePooling1D	2	1	2	1496 × 16
Block_2	conv1d_3	Conv1D	5	32	1	1492 × 32
	batch normalization_3	Batch Normalization	-	-	-	1492 × 32
	conv1d_4	Conv1D	5	32	1	1488 × 32
	batch normalization_4	Batch Normalization	-	-	-	1488 × 32
	average_pooling1d_2	AveragePooling1D	2	1	2	744 × 32
Block_3	conv1d_5	Conv1D	5	32	1	740 × 32
	batch normalization_5	Batch Normalization	-	-	-	740 × 32
	global_average_pooling1d	GlobalAveragepooling	-	-	-	32
	dense_1	Dense	-	-	-	32
	dense_2	Dense	-	-	-	5

Hyperparameters: optimizer = Adam, batch size = 128, learning rate = 0.001 (reduce_lr: min_lr = 0.0001).

**Table 3:** Performance of the recordwise and subjectwise training approaches.

**Record-Wise Training**				
	**Accuracy (%)**	**Sensitivity (%)**	**Specificity (%)**	**Time**
preictal 20–10	99.37 (±0.14%)	99.47 (±024%)	99.27 (±047%)	2 h 12 min 43 s
preictal 30–20	98.61 (±0.20%)	98.21 (±0.12%)	99.03 (±0.35%)	2 h 13 min 43 s
preictal 40–30	99.59 (±0.22%)	99.77 (±0.13%)	99.40 (±0.44%)	2 h 04 min 06 s
**Subject-** **Wise** **Training**				
	**Accuracy (%)**	**Sensitivity (%)**	**Specificity (%)**	**Time**
preictal 20–10	84.25 (±0.20%)	82.45 (±1.39%)	82.45 (±1.39%)	2 h 17 min 07 s
preictal 30–20	84.46 (±0.20%)	84.81 (±0.94%)	84.12 (±1.09%)	2 h 19 min 11 s
preictal 40–30	86.17 (±0.84%)	88.73 (±0.90%)	83.60 (±2.05%)	2 h 20 min 03 s

**Table 4:** Classification of accuracy, sensitivity, and specificity (mean values) of the patient-specific interictal and preictal classification transfer learning models.

NO.	# of Frozen Layers	Preictal 20–10				Preictal 30–20				Preictal 40–30			
		Acc (%)	Sen (%)	Spe (%)	Time (s)	Acc (%)	Sen (%)	Spe (%)	Time (s)	Acc (%)	Sen (%)	Spe (%)	Time (s)
2	3	98.0	96.1	100	42	100	100	100	39	99.5	100	99.1	43
	6	100	100	100	40	100	100	100	37	100	100	100	37
	9	100	100	100	35	100	100	100	46	100	100	100	35
	12	97.5	100	94.7	104	97.0	93.3	100	112	100	100	100	107
4	3	100	100	100	42	100	100	100	41	100	100	100	43
	6	100	100	100	37	100	100	100	37	100	100	100	35
	9	100	100	100	35	100	100	100	46	100	100	100	33
	12	94.9	90.4	100	107	100	100	100	118	97.5	100	95.4	109
5	3	100	100	100	39	100	100	100	37	100	100	100	33
	6	100	100	100	34	100	100	100	34	100	100	100	31
	9	100	100	100	43	100	100	100	30	100	100	100	28
	12	100	100	100	107	100	100	100	109	100	100	100	103
7	3	100	100	100	39	100	100	100	36	100	100	100	42
	6	100	100	100	36	100	100	100	32	100	100	100	35
	9	100	100	100	44	100	100	100	33	100	100	100	32
	12	100	100	100	109	100	100	100	109	100	100	100	109
8	3	100	100	100	41	100	100	100	56	100	100	100	37
	6	100	100	100	32	100	100	100	33	100	100	100	36
	9	100	100	100	28	100	100	100	29	100	100	100	36
	12	100	100	100	109	100	100	100	109	99.5	100	97.7	109
9	3	100	100	100	35	99.5	100	99.33	37	100	100	100	49
	6	100	100	100	37	100	100	100	35	100	100	100	35
	9	100	100	100	33	100	100	100	34	100	100	100	31
	12	100	100	100	108	97.5	100	96.66	100	100	100	100	109
10	3	100	100	100	46	100	100	100	44	100	100	100	46
	6	100	100	100	34	100	100	100	40	100	100	100	36
	9	100	100	100	33	100	100	100	33	100	100	100	32
	12	97.5	94.7	100	89	100	100	100	109	100	100	100	109
11	3	100	100	100	40	97.5	94.11	100	41	99.5	100	98.57	36
	6	100	100	100	31	99	97.64	100	38	99.5	99.23	100	33
	9	100	100	100	30	98.99	100	98.6	33	97.5	96.15	100	29
	12	100	100	100	109	94.99	88.23	100	109	97.5	96.1	100	109
13	3	100	100	100	48	98	95.7	100	36	100	100	100	34
	6	100	100	100	36	99.9	98.9	100	28	100	100	100	36
	9	100	100	100	30	100	100	100	28	100	100	100	29
	12	100	100	100	109	100	100	100	105	100	100	100	106

**Table 5:** Classification accuracy, Cohen’s kappa, and F1 score (mean ± standard deviation) of the EEG model, ECG model, and the EEG–ECG transfer learning model.

Model	Accuracy	Kappa	F1	Time
EEG	92.67 (±0.45%)	0.908 (±0.006)	92.69 (±0.45%)	1 h 32 min 42 s
ECG	86.13 (±1.49%)	0.827 (±0.019)	86.07 (±1.46%)	1 h 38 min 10 s
EEG–ECG (frozen block_1)	88.64 (±1.00%)	0.858 (±0.013)	88.59 (±1.01%)	47 min 31 s
EEG–ECG (frozen block_1&2)	82.16 (±0.56%)	0.777 (±0.007)	82.12 (±0.52%)	17 min 00 s
EEG–ECG (frozen block_1~3)	63.38 (±0.62%)	0.542 (±0.008)	63.19 (±0.60%)	17 min 05 s

**Table 6:** Performance of different seizure prediction systems based on CNNs with EEG signals.

Study	Dataset	Input	Model	Training Type	Acc (%)	Sen (%)	Spe (%)
Dissanayake et al. [30]	Siena EEG	MFCCs	C-GNN (distance-based)	S-Ind	96.0	96.0	96.6
			C-GNN (partially learned)		95.5	95.1	95.1
Zhao et al. [31]	CHB-MIT	Raw data	1D-CNN	P-Spc	-	88.7	-
			ResCNN			89.9	
			SCL-AddNets			93	
This Study	CHB-MIT	Raw data (GFP)	1D-CNN + transfer learning	P-Spc	99.73	99.79	99.65
	Siena EEG + Zenodo	Raw data (GFP)	1D-CNN + transfer learning	P-Spc	99.9	99.9	100

S-Ind: subject independent; P-Spc: patient-specific.

**Table 7:** Performance of different sleep staging systems based on CNNs with EEG signals.

Study	Dataset	Input	Model	# CNN Layer	Sleep Stage	Acc (%)	Kappa	F1 (%)
Li et al. [38]	sleep-edfx	Spectrogram	EEGSNet	15	Wake-REM-N1-N2-N3	83.02	0.770	77.26
Jadhav et al. [24]	sleep-edfx	Raw data	1D-CNN	6	Wake-REM-N1-N2-N3	83.59	0.780	77.00
		SWT	2D-CNN	6		85.49	0.800	78.70
		STFT	2D-CNN	4		85.81	0.800	79.70
This Study	sleep-edfx	Raw data	1D-CNN	5	Wake-REM-N1-N2-N3	92.67	0.908	92.69

**Table 8:** Performance of different sleep staging systems based on CNNs with ECG signals.

Study	Dataset	Input	Model	# Class	Sleep Stages	Acc (%)	Kappa	F1 (%)
Urtnasan et al. [8]	Samsung Medical Center	Raw data	CNN+GRU	3	Wake-NREM-REM	86.40	-	-
				5	Wake-REM-N1-N2-N3	74.20	-	-
Tang et al. [36]	SHHS2	Raw data	CNN+GRU (Domain adaptation)	4	Wake-REM- Light-Deep	78.70	0.749	-
	SHHS1					74.80	0.675	-
	MESA					80.60	0.705	-
This Study	HMC sleep center	Raw data	1D-CNN (ECG)	5	Wake-REM-N1-N2-N3	86.13	0.827	86.07
			1D-CNN (EEG-ECG)			88.64	0.858	88.59

## Data Availability

The data used in this study are openly available in Siena scalp EEG database at [https://physionet.org/content/siena-scalp-eeg/1.0.0/ accessed on 10 February 2022], Zenodo at [https://zenodo.org/record/1415495 accessed on 10 February 2022], Sleep-EDF expanded database at [https://physionet.org/content/sleep-edfx/1.0.0/ accessed on 10 February 2022], and Haaglanden Medisch Centrum sleep staging database at [https://physionet.org/content/hmc-sleep-staging/1.0.1/ accessed on 10 February 2022].

## References

[1] Binnie C.D., Prior P.F. (1994). Electroencephalography. J. Neurol. Neurosurg. Psychiatry Res..

[2] Oh S.L., Hagiwara Y., Raghavendra U., Yuvaraj R., Arunkumar N., Murugappan M., Acharya U.R. (2018). A deep learning approach for Parkinson’s disease diagnosis from EEG signals. Neural. Comput. Appl..

[3] Rim B., Sung N.J., Min S., Hong M. (2020). Deep learning in physiological signal data: A survey. Sensors.

[4] Han C., Peng F., Chen C., Li W., Zhang X., Wang X., Zhou W. (2021). Research progress of epileptic seizure predictions based on electroencephalogram signals. J. Biomed. Eng..

[5] Yang C.Y., Huang Y.Z. (2022). Parkinson’s Disease Classification Using machine learning approaches and resting-state EEG. J. Med. Biol. Eng..

[6] Shoeibi A., Sadeghi D., Moridian P., Ghassemi N., Heras J., Alizadehsani R., Khadem A., Kong Y., Nahavandi S., Zhang Y. (2021). Automatic diagnosis of schizophrenia in EEG signals using CNN-LSTM models. Front. Neurosci..

[7] Cimtay Y., Ekmekcioglu E. (2020). Investigating the use of pretrained convolutional neural network on cross-subject and cross-dataset EEG emotion recognition. Sensors.

[8] Urtnasan E., Park J.U., Joo E.Y., Lee K.J. (2022). Deep convolutional recurrent model for automatic scoring sleep stages based on single-lead ECG signal. Diagnostics.

[9] Panigrahi S., Nanda A., Swarnkar T. (2021). A Survey on Transfer Learning. Intelligent and Cloud Computing.

[10] Phan H., Chén O.Y., Koch P., Lu Z., McLoughlin I., Mertins A., De Vos M. (2021). Towards more accurate automatic sleep staging via deep transfer learning. IEEE Trans. Biomed. Eng..

[11] Wan Z., Yang R., Huang M., Zeng N., Liu X. (2021). A review on transfer learning in EEG signal analysis. Neurocomputing.

[12] Zargar B.S., Mollaei M.R.K., Ebrahimi F., Rasekhi J. (2023). Generalizable epileptic seizures prediction based on deep transfer learning. Cogn. Neurodyn..

[13] Bird J.J., Kobylarz J., Faria D.R., Ekárt A., Ribeiro E.P. (2020). Cross-domain MLP and CNN transfer learning for biological signal processing: EEG and EMG. IEEE Access.

[14] Moshe S.L., Perucca E., Ryvlin P., Tomson T. (2015). Epilepsy: New advances. Lancet.

[15] Lun X., Yu Z., Chen T., Wang F., Hou Y. (2020). A Simplified CNN Classification Method for MI-EEG via the Electrode Pairs Signals. Front. Hum. Neurosci..

[16] Goldberger A.L., Amaral L.A., Glass L., Hausdorff J.M., Ivanov P.C., Mark R.G., Mietus J.E., Moody G.B., Peng C.K., Stanley H.E. (2000). PhysioBank, PhysioToolkit, and PhysioNet: Components of a new research resource for complex physiologic signals. Circulation.

[17] Detti P., Vatti G., Zabalo M.D.L. (2020). EEG Synchronization Analysis for seizure prediction: A study on data of noninvasive recordings. Processes.

[18] Billeci L., Marino D., Insana L., Vatti G., Varanini M. (2020). Patient-specific seizure prediction based on heart rate variability and recurrence quantification analysis. PLoS ONE.

[19] Skrandies W. (1989). Data reduction of multichannel fields: Global field power and principal component analysis. Brain Topogr..

[20] Wang X., Wang X., Liu W., Chang Z., Kärkkäinen T., Cong F. (2021). One dimensional convolutional neural networks for seizure onset detection using long-term scalp and intracranial EEG. Neurocomputing.

[21] Kemp B., Zwinderman A.H., Tuk B., Kamphuisen H.A., Oberyé J.J. (2000). Analysis of a sleep-dependent neuronal feedback loop: The slow-wave microcontinuity of the EEG. IEEE Trans. Biomed. Eng..

[22] Alvarez-Estevez D., Rijsman R.M. (2021). Inter-database validation of a deep learning approach for automatic sleep scoring. PLoS ONE.

[23] Hori T., Sugita Y., Koga E., Shirakawa S., Inoue K., Uchida S., Kuwahara H., Kousaka M., Kobayashi T., Tsuji Y. (2001). Proposed supplements and amendments to ‘A Manual of Standardized Terminology, Techniques and Scoring System for Sleep Stages of Human Subjects’, the Rechtschaffen & Kales (1968) standard. Psychiatry Clin..

[24] Jadhav P., Mukhopadhyay S. (2022). Automated Sleep Stage Scoring Using Time-frequency spectra convolution neural network. IEEE Trans. Instrum. Meas..

[25] Acharya U.R., Oh S.L., Hagiwara Y., Tan J.H., Adeli H. (2018). Deep convolutional neural network for the automated detection and diagnosis of seizure using EEG signals. Comput. Biol. Med..

[26] Wei X., Zhou L., Zhang Z., Chen Z., Zhou Y. (2019). Early prediction of epileptic seizures using a long-term recurrent convolutional network. J. Neurosci..

[27] Wang Y., Yang Y., Cao G., Guo J., Wei P., Feng T., Dai Y., Huang J., Kang G., Zhao G. (2022). SEEG-Net: An explainable and deep learning-based cross-subject pathological activity detection method for drug-resistant epilepsy. Comput. Biol. Med..

[28] Al-Kadi M.I., Reaz M.B.I., Ali M.A.M. (2013). Evolution of electroencephalogram signal analysis techniques during anesthesia. Sensors.

[29] Ardalan Z., Subbian V. (2022). Transfer learning approaches for neuroimaging analysis: A scoping review. Front. Artif. Intell..

[30] Dissanayake T., Fernando T., Denman S., Sridharan S., Fookes C. (2022). Geometric Deep learning for subject independent epileptic seizure prediction using scalp EEG signals. IEEE J. Biomed. Health Inform..

[31] Zhao Y., Li C., Qian R., Song R., Chen X. (2022). Patient-specific seizure prediction via adder nNetwork and supervised contrastive learning. IEEE Trans. Neural. Syst. Rehabil. Eng..

[32] da Silveira T.L., Kozakevicius A.J., Rodrigues C.R. (2017). Single-channel EEG sleep stage classification based on a streamlined set of statistical features in wavelet domain. Med. Biol. Eng. Comput..

[33] Yildirim O., Baloglu U.B., Acharya U.R. (2019). Deep learning model for automated sleep stages classification using PSG signals. Int. J. Environ. Res. Public Health.

[34] Ebrahimi F., Setarehdan S.K., Nazeran H. (2015). Automatic sleep staging by simultaneous analysis of ECG and respiratory signals in long epochs. Biomed. Signal. Process. Control.

[35] Wei Y., Qi X., Wang H., Liu Z., Wang G., Yan X. (2019). A multi-class automatic sleep staging method based on long short-term memory network using single-lead electrocardiogram signals. IEEE Access.

[36] Tang M., Zhang Z., He Z., Li W., Mou X., Du L., Wang P., Zhao Z., Chen X., Li X. (2022). Deep adaptation network for subject-specific sleep stage classification based on a single-lead ECG. Biomed. Signal. Process. Control.

[37] Radha M., Fonseca P., Moreau A., Ross M., Cerny A., Anderer P., Long X., Aarts R.M. (2021). A deep transfer learning approach for wearable sleep stage classification with photoplethysmography. NPJ Digit. Med..

[38] Li C., Qi Y., Ding X., Zhao J., Sang T., Lee M. (2022). A deep learning method approach for sleep stage classification with EEG spectrogram. Int. J. Environ. Res. Public Health.

